# Detargeting Lentiviral-Mediated CFTR Expression in Airway Basal Cells Using miR-106b

**DOI:** 10.3390/genes11101169

**Published:** 2020-10-06

**Authors:** Soon H. Choi, Rosie E. Reeves, Guillermo S. Romano Ibarra, Thomas J. Lynch, Weam S. Shahin, Zehua Feng, Grace N. Gasser, Michael C. Winter, T. Idil Apak Evans, Xiaoming Liu, Meihui Luo, Yulong Zhang, David A. Stoltz, Eric J. Devor, Ziying Yan, John F. Engelhardt

**Affiliations:** 1 Department of Anatomy and Cell Biology, University of Iowa, Carver College of Medicine, Iowa City, IA 52242, USA; soon-choi@uiowa.edu (S.H.C.); rosiereeves10@gmail.com (R.E.R.); tom-lynch@outlook.com (T.J.L.); weam-shahin@uiowa.edu (W.S.S.); zehua-feng@uiowa.edu (Z.F.); grace-gasser@uiowa.edu (G.N.G.); michael-winter@uiowa.edu (M.C.W.); idil-apak@uiowa.edu (T.I.A.E.); xiaoming-liu@uiowa.edu (X.L.); meihui-luo@uiowa.edu (M.L.); yulong-zhang@uiowa.edu (Y.Z.); ziying-yan@uiowa.edu (Z.Y.); 2 Molecular Medicine Program, University of Iowa, Carver College of Medicine, Iowa City, IA 52246, USA; guillermo-romanoibarra@uiowa.edu; 3 Department of Internal Medicine, University of Iowa, Carver College of Medicine, Iowa City, IA 52246, USA; david-stoltz@uiowa.edu; 4 Department of Obstetrics and Gynecology, University of Iowa, Carver College of Medicine, Iowa City, IA 52246, USA; eric-devor@uiowa.edu

**Keywords:** miRNA, airway basal cell, CFTR, gene therapy, lentivirus

## Abstract

Lentiviral-mediated integration of a *CFTR* transgene cassette into airway basal cells is a strategy being considered for cystic fibrosis (CF) cell-based therapies. However, *CFTR* expression is highly regulated in differentiated airway cell types and a subset of intermediate basal cells destined to differentiate. Since basal stem cells typically do not express CFTR, suppressing the *CFTR* expression from the lentiviral vector in airway basal cells may be beneficial for maintaining their proliferative capacity and multipotency. We identified miR-106b as highly expressed in proliferating airway basal cells and extinguished in differentiated columnar cells. Herein, we developed lentiviral vectors with the miR-106b-target sequence (miRT) to both study miR-106b regulation during basal cell differentiation and detarget CFTR expression in basal cells. Given that miR-106b is expressed in the 293T cells used for viral production, obstacles of viral genome integrity and titers were overcome by creating a 293T-B2 cell line that inducibly expresses the RNAi suppressor B2 protein from flock house virus. While miR-106b vectors effectively detargeted reporter gene expression in proliferating basal cells and following differentiation in the air–liquid interface and organoid cultures, the CFTR-miRT vector produced significantly less CFTR-mediated current than the non-miR-targeted CFTR vector following transduction and differentiation of CF basal cells. These findings suggest that miR-106b is expressed in certain airway cell types that contribute to the majority of CFTR anion transport in airway epithelium.

## 1. Introduction

Cystic fibrosis (CF) is an inherited disease caused by mutations in the cystic fibrosis transmembrane conductance regulator (*CFTR*) gene [1]. *CFTR* is expressed primarily in epithelial cells of multiple organs. CFTR plays an important role in transepithelial anion transport important for regulating airway surface fluid volume, viscosity, and pH [2]. Lung disease with CF involves thick viscous mucus and chronic bacterial infections and is the primary cause of mortality. Gene and cell-based therapies for CF lung disease are gaining momentum, but knowledge gaps do remain regarding the target airway cell types that can prevent or reverse lung disease once a functional *CFTR* gene is expressed [3]. 

Both the proximal and distal airways express CFTR, but the landscape of cell types and CFTR expression patterns differ in these two levels of the airway. In the proximal airways, basal cells are considered the major stem cell precursor for ciliated cells, goblet cells, ionocytes, and other specialized cell types [3,4]. *CFTR* is expressed at widely divergent levels in a subset of proximal airway basal cells, secretory (goblet) cells, and ionocytes [5,6]. In the distal airway, basal and club cells are generally considered multipotent or bipotent stem cells, respectively, and can both give rise to ciliated cells. CFTR is most abundantly expressed in club secretory cells of bronchioles and alveolar type II cells [3,7,8].

Delivery of the *CFTR* gene to the CF airway basal cell is of particular interest in CF cell-based therapies, as this stem cell target has the ability to self-renew and differentiate into secretory cells (goblet or club), ciliated cells, and ionocytes. Lentiviral vectors have advantages over other widely used gene delivery vectors, such as adeno-associated vector (AAV), because lentiviruses integrate into the host genome and persist following cell division. However, CFTR is not typically expressed in multipotent airway basal cells but is rather expressed in transitional (intermediate) basal cells fated to become secretory cells [3,6,7]. Given that the functional role of CFTR expression in basal cell differentiation is unknown, methods to regulate transgene-derived CFTR expression in multipotent and transitional basal cell states and mimic endogenous patterns of expression could provide greater efficacy in CF cell therapy approaches. 

We hypothesized that this pattern of expression could be achieved by suppressing *CFTR* expression in multipotent basal cells via miRNA-mediated silencing. This approach of suppressing transgene expression in a specific cell type is most often referred to as “detargeting”. To this end, we sought to identify a miRNA that was selectively expressed in multipotent basal cells and identified miR-106b. The target sequence of miR-106b was then incorporated into the 3′-untranslated region (UTR) of reporter and *CFTR* transgene cassettes encoded within bicistronic and bidirectional lentiviral vectors. Here, we describe the challenges and solutions for vector production using this approach, the analysis of dual reporter gene vectors that demonstrate the efficiency of basal cell detargeting of transgene expression, and the functional consequences of downregulating *CFTR* expression in CF human basal cells by assessing their capacities for generating CFTR currents following differentiation. We believe these vectors created will provide new opportunities for studying pathways that control lineage-commitment of airway basal cells, understanding cell type-specific functions of CFTR function, and ultimately aid in developing more effective gene therapy approaches for CF.

## 2. Materials and Methods 

### 2.1. Proviral Vector Plasmid Construction

pLV-dt/EGFP is a proviral lentiviral transfer plasmid. It is derived from pLent6/V5-GW/lacZ (Invitrogen) by inserting a phosphoglycerate kinase 1 promoter (PGK) driven dTomato expression cassettes (PGK-dTomato or dt) in the same direction as the lentiviral genomic transcript and a human cytomegalovirus enhancer beta-actin promoter (CBA) driven nuclear EGFP expression cassettes (CBA-EGFP) in opposite orientations. The EGFP reporter has the SV40 large T antigen nuclear localization signal sequence attached to its C-terminus.

pLV-dt/ΔEGFP is derived from pLV-dt/EGFP following deletion of the CBA promoter. This vector was used to confirm that enhanced titers generated in the presence of B2 was due to antisense transcripts derived from the CBA-EGFP expression cassette.

pLV-dt/EGFP-miRT and pLV-dt/EGFP-RmiRT are derivates of pLV-dt/EGFP. pLV-dt/EGFP-miRT has four tandem 21-nt long sequences complementary to miR-106b (4× miR-target or miRT) within the 3’ UTR of the CBA-EGFP cassette. pLV-dt/EGFP-RmiRT is the control vector with the miRT sequences placed in the reverse orientation (reverse-4× miRT or RmiRT) within the 3′ UTR of the CBA-EGFP cassette.

pLV-dt/CFTR-miRT was constructed by deleting the CBA-EGFP cassette from pLV-dt/EGFP-miRT but leaving the miRT within the vector. pLV-dt/CFTR-Ø was constructed by deleting both the CBA-EGFP and cassette from pLV-dt/EGFP-miRT. Subsequently, the PGK-CFTR fragment was cloned in the opposite orientation to the Tomato cassette and the CBA promoter placed in front of the Tomato transgene cassette.

TripZ-B2 was constructed using a binary plant vector pCassRZ containing FHV RNA1 cDNA (a generous gift from Jang-Kyun Seo and ALN Rao) as template for amplifying B2 by PCR using a 5′ forward primer encoding an AgeI site (underlined) (5′-AAAAAAACCGGTGCCGCCACCATGCCAAGCAAACTCGCGCTAATCC-3′) and a 3′ reverse primer encoding a MluI site (underlined) (5′-AAAAAAACGCGTTTTCGGGCTAGAACGGGTGTGGGTG-3′). The resulting B2 gene PCR product was digested with AgeI and MluI, and subcloned into TripZ vector (Thermo Scientific) under the control of tetracycline response element.

All lentiviral vector plasmids were amplified by transforming Stbl3 competent *Escherichia coli* (*E. coli*.) (ThermoFisher Scientific, #C7373, Waltham, MA, USA). DNA purification was carried out using QIAprep Miniprep kits (QIAGEN, #27104, Hilden, Germany) and Nucleobond Xtra Maxi EF kits (Takara, #740414, Kusatsu, Japan). All vector plasmids were Sanger sequenced to confirm integrity.

### 2.2. Cell Culture and Human Basal Expansion

The human embryonic kidney cell line HEK293T was used for vector production and cultured in Dulbecco’s modified Eagle’s medium (DMEM) with 10% fetal bovine serum (FBS) and 1% Penicillin/Streptomycin (P/S). Primary human airway epithelial cells were isolated from the dissected tracheobronchial airway of CF (ΔF508/G551D) and non-CF lungs obtained at the time of lung transplantation and were obtained from the Cells and Tissue Core at the University of Iowa Carver College of Medicine. When lentivirus transduced basal cell cultures were expanded for FACS isolation, they were cultured under dual SMAD signaling inhibition using Small Airway Epithelial Growth Medium (SAGM; Lonza, #CC-3118, Basel, Switzerland) supplemented with extra additives (SAGM-EA) on tissue culture plates precoated with Collagen IV (Sigma, #C7521, St. Louis, MO, USA), as previously described [9]. For experiments that used unsorted populations of lentivirus transduced human basal cells passaged only 2–3 times, cells were cultured in Bronchial Epithelial Cell Growth Medium BulletKit (BEGM; Lonza, #CC-3170, Basel, Switzerland) and directly seeded onto a transwell filter culture at an air–liquid interface.

### 2.3. Generation of Differentiated Air–Liquid Interface Cultures

Polarized human airway epithelial cultures were generated at an air–liquid interface by seeding 2 × 10^5^ basal cells onto transwell inserts with polyester membrane (Corning, #3450, Corning, NY, USA) that was precoated with collagen IV (Sigma #C7521, St. Louis, MO, USA). Seeding occurred in SAGM-EA or BEGM, depending on the experimental design, and at 24 h post-seeding the basal cell culture medium was replaced with PneumaCult ALI medium (StemCell Technologies, Vancouver, Canada) in both the apical and basal chambers. The next day, the apical chamber media was aspirated, and the basal chamber media was replaced every other day for a minimum of 21 days before analysis. 

### 2.4. microRNA Inhibitor Transfection

Anti-miR miRNA inhibitor for has-miR-106b (ThermoFisher Scientific, Assay ID AM10067, #AM17000, Waltham, MA, USA) and anti-miR miRNA inhibitor negative Control #1 (ThermoFisher Scientific, #AM17010, Waltham, MA, USA) were used to transfect the 293T cells transduced with LV-dt/EGFP-miRT. The transfection procedure followed the RNAi transfection protocol provided with Lipofectamine RNAiMAX Transfection Reagent (ThermoFisher Scientific, #13778100, Waltham, MA, USA). 

### 2.5. Lentiviral Vector Production

Lentiviral vector production was performed using a previously published protocol [10] with slight modifications in 293T and 293T-B2 cells. When the 293T-B2 cells were used, doxycycline was added at the time of Ca_2_PO_4_ transfection (500 ng/mL) with viral production vector: pMD2.G (VSV-G envelope expressing vector), psPAX2 (packaging vector) and the proviral vector plasmid (pLV-dt/EGFP-miRT, pLV-dt/EGFP-RmiRT, pLV-dt/CFTR-miRT, or pLV-dt/CFTR-Ø). At ~12–16 h post-transfection, the medium was changed to DMEM with 2% FBS. At 24 h and 48 h after the first medium change, the medium containing lentivirus is harvested and filtered (0.4 μm pore size). The virus was concentrated ~100-fold using a Lenti-X Concentrator (Takara, #631232, Kusatsu, Japan) and then resuspended in a medium of choice. Lentiviral vector titers were calculated by serial dilution on 293T cells followed by flow cytometry for Tomato expression at 3 days post-infection, as previously described [10]. 

### 2.6. Creation of the Doxycycline-Inducible 293T-B2 Cell Line

The TripZ-B2 plasmid described above was used to produce a lentiviral vector for transduction of 293T cells. The virally transduced cells were selected with puromycin treatment (3 μg/mL) for 5 days. After that, 0.25 μg/mL of puromycin was used for 293T-B2 maintenance and expansion. B2 was induced by addition of doxycycline to the culture medium (Sigma, #D9891), as described above. 

### 2.7. qPCR miRNA Arrays

Total RNA was extracted using the miRVana miRNA isolation kit (Ambion, #AM1560, Austin, TX, USA). RNA quality and concentrations were analyzed on a NanoDrop M-1000 spectrophotometer and an Agilent 2100 Bioanalyzer. RNAs with quality scores >7.00 were used for expression assays. RNA concentrations were standardized to 200 ng/μL. TaqMan low-density miRNA arrays (TLDAs) (Applied Biosystems, #4444913, Foster City, CA, USA) were used to assess miRNA expression levels in proliferating basal cells grown in SAGM-EA. Reverse transcription of 600 ng total RNA was carried out using a TaqMan miRNA reverse transcriptase kit (Applied Biosystems, #4366596) with Megaplex RT primers, Human Pool (Applied Biosystems, #4399966). Samples were loaded onto the TLDA, which utilizes 384 wells preloaded with specific miRNA probes and primers in each well. The TLDA data were processed on an Applied Biosystems Model 7900 Genetic Analyzer, and the data were analyzed using the Applied Biosystems StatMiner software. Each sample was analyzed in triplicate, and each Ct value was normalized to the Ct value of RNU48 endogenous RNA control. Relative quantification of each miRNA was performed using the ΔΔCt method. Statistical significance of the fold change was assessed using two-tailed t-tests. *p*-values of <0.05 were taken as statistically significant.

### 2.8. Quantitative Real-Time PCR of miRNAs

TaqMan miRNA assays for homo sapiens (has) miR-106b, miR-25, miR-93, and RNU-48 are from ThermoFisher′s MicroRNA Analysis products (#4427975), and their Assay IDs are 000442, 000403, 000432, and 001006, respectively. The qPCR was performed according to their protocol (thermofisher.com/taqmanfiles, Waltham, USA).

### 2.9. Transduction of Human Primary Airway Basal Cells

Primary human basal cells were plated on 6-well plates at 25%–30% confluence for lentivirus infection in the presence of DEAE-Dextran (6 μg/mL) [11]. On the day following plating, the lentiviral vector solution was mixed with culture medium (2 mL total volume with ~5 × 10^6^ transduction units (TU)) and added to each well and incubated overnight before the medium was changed. Typically, the level of transduction based on Tomato expression was 30–50% of cells.

### 2.10. Organoid Culture

The membranes of 24-well transwells (Corning) were coated with 20 μL of a 1:1 PneumaCult-ALI:cold Matrigel (Corning, #354277) mixture and then incubated at 37 °C for 30 min. The airway basal cells (~11,000 cells/well) in the medium and cold Matrigel are mixed at a 1:1 (*v*/*v*) ratio and 50 μL of the Matrigel/cell mixture was applied onto the transwell. After incubation at 37 °C for 30 min, PneumaCult-ALI (StemCell Technologies, #05001) was added on top of the Matrigel in the apical chamber and basal chamber. The medium was then changed every other day and the organoids were analyzed after ~3 weeks by staining with Hoescht 33342 (10 μg/mL) for one hour and imaged live on a confocal microscope (LSM 880, Zeiss, Oberkochen, Germany).

### 2.11. Immunohistochemistry and Microscopy

ALI membranes were fixed in 4% paraformaldehyde (PFA) overnight prior to washing with phosphate-buffered saline (PBS) and embedding in Tissue-Tek^®^ O.C.T. Compound (OCT) frozen blocks. Frozen sections were cut at 10 μm and post-fixed in 4% PFA for 20 min, rinsed three times with PBS, and then incubated in blocking buffer containing 20% donkey serum, 0.5% triton X-100, 1 mM CaCl_2_ in PBS for 1 h. Samples were then blocked with 1% donkey serum and then incubated with primary antibody in diluent buffer containing 1% donkey serum, 0.5% triton X-100 and 1 mM CaCl_2_ in PBS overnight at 4 °C. Slides were then washed twice with PBS and then incubated with secondary antibody in diluent buffer at room temperature for 1 h. Nuclei were stained with Hoescht 33342 (10 μg/mL). The primary antibodies were chicken anti-GFP (1:1000, Aves Lab, #GFP-1020) and rabbit anti-keratin 5 (1:500, BioLegend, #PRB160P). The secondary antibodies used were Alexa Fluor 488 labeled donkey anti-chicken IgG (1:250, Jackson ImmunoResearch, #703-546-155, West Grove, PA, USA) and Alexa Fluor 647 labeled donkey anti-rabbit IgG (1:250, Jackson ImmunoResearch, #711-606-152, West Grove, PA, USA). Slides were washed three times with PBS and then mounted with Aquamount (Thermo Scientific, VWR #41799-008, Waltham, MA, USA). Images of stained slides were obtained using an LSM 880 confocal microscope (Zeiss, Oberkochen, Germany). 

### 2.12. Flow Cytometry

We used fluorescence-activated cell sorting (FACS) to isolate pure populations of Tomato-positive basal cells from LV-dt/EGFP-RmiRT and LV-dt/EGFP-miRT transduced cultures. These cells were then seeded into ALI cultures and differentiated for 21 days. Cells were then dissociated with Accutase (StemCell Technologies, #07920), centrifuged at 200 RCM for 5 min, and resuspended in 1mL PBS without calcium or magnesium chloride. To evaluate EGFP expression in various cell types, cells were fixed and permeabilized using the Foxp3 Fixation/Permeabilization kit following the manufacturer’s protocol (eBiosciences/ThermoFisher #005523-00, Waltham, MA, USA). Cells were stained with the following antibodies: BSND (Abcam clone EPR14270, Cambridge, UK), MUC5AC (Novus clone 45M1, Littleton, CO, USA), acetylated alpha tubulin (Cell Signaling clone D20G3 conjugated to Alexa 647), p63 (Abcam clone EPR5701 conjugated to Alexa647). BSND and MUC5AC were stained with goat anti-rabbit and goat anti-mouse polyclonal antibodies conjugated to Alexa 647 (Invitrogen/ThermoFisher; #A-21244 and #A21235, Waltham, MA, USA). Stained cells were then run on an Attune N×T Flow Cytometer (ThermoFisher, Waltham, MA, USA) and analyzed using FlowJo version 10.7 (Ashland, OR, USA). 

### 2.13. Short-Circuit Current Measurements

Short-circuit currents were measured in CF ALI cultures generated from LV-dt/EGFP-RmiRT and LV-dt/EGFP-miRT transduced basal cells following differentiation for at least 3 weeks. Transwells were placed under VCC MC8 voltage clamps and P2300 Ussing chambers (Physiologic Instruments, San Diego, CA, USA) with low chloride buffer in the apical chamber and high chloride buffer in the basal chamber, as previously described [12,13]. The change in current was assessed after the sequential addition of the following antagonists and agonists: 100 μM amiloride (ENaC inhibitor), 100 μM 4,4′-Diisothiocyano-2,2′-stilbenedisulfonic acid (DIDS) (a general chloride channel blocker that does not affect CFTR), 100 μM 3-Isobutyl-1-methylxanthine (IBMX) and 10 μM forskolin (to increase intracellular cAMP levels which activate CFTR), and 50 μM GlyH101 (a CFTR channel blocker).

### 2.14. Statistical Analysis

Statistical analysis and graphical presentation were performed using Microsoft Excel (version 16.41, Redmond, WA, USA), GraphPad Prism (version 8, San Diego, CA, USA), and RStudio (version 1.3.959, Boston, MA, USA). Statistical significance in the TLDA data was analyzed using Student’s *t*-test without assuming a consistent standard deviation between genes and adjusted for multiple comparisons using a false discovery rate approach using a two-stage linear step-up procedure of Benjamini, Krieger and Yekutieli, with Q = 5%. Correlation of miRNA expression between passage 3 and 18 was tested using linear regression analysis in RStudio (version 1.3.959, Boston, MA, USA). One-way ANOVA and Bonferroni’s multiple comparisons test were used for lentiviral vector titration. One-way ANOVA and Tukey’s multiple comparisons test were used for Isc analysis and qPCR. One-way ANOVA and Dunnett’s multiple comparisons test were used for cell type analysis flow cytometry.

## 3. Results

### 3.1. Basal Cells Stably Express miR-106b in Conditional Reprogramming Proliferative Cultures for Long-Term Culture 

**Figure 1:**
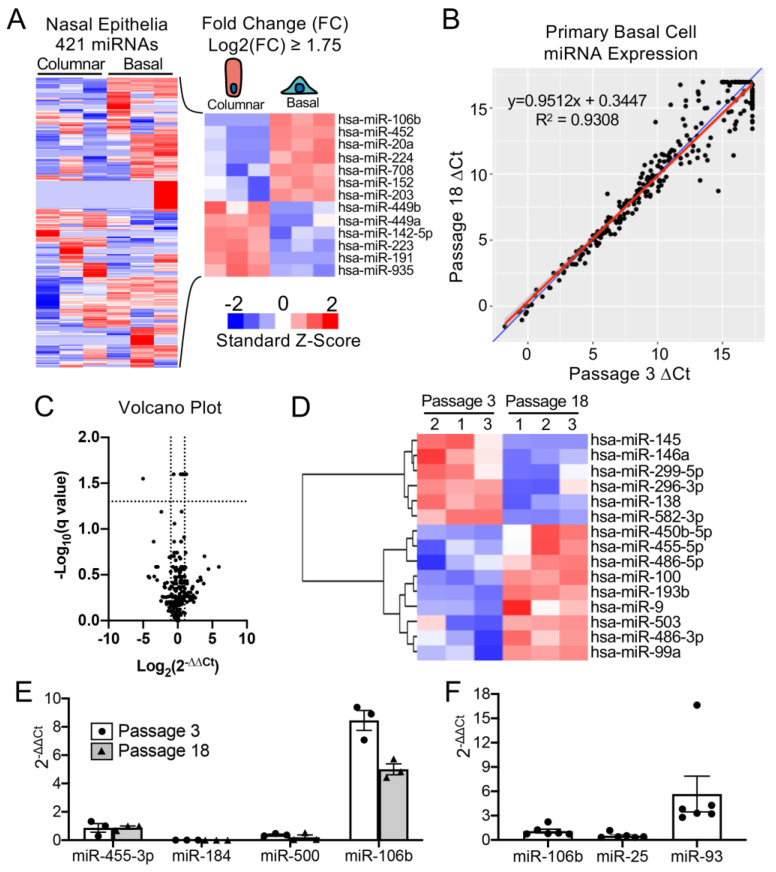
mMiR-106b is stably expressed at high levels in proliferating human basal cells. (**A**) Published data of miRNAs detected by high throughput sequence profiling of nasal basal cells and columnar cells (Accession: GSE22145) were used to generate a heatmap of 421 expressed miRNAs (left) and 13 miRNAs with a Log2 fold difference greater than 1.75 or less than −1.75 (right). (**B**) Correlation of miRNA expression in basal cells at Passage 3 and Passage 18 detected by qPCR array with the blue line represents a theoretical perfect correlation (x = y), and the red line represents linear regression model. (**C**) Volcano plot of miRNA array data indicating genes that were differentially expressed between passages 3 and 18. (**D**) Heatmap of miRNA array data with unsupervised hierarchical clustering of 15 miRNAs (of 252 detected) with an absolute fold change ≥ 2 and an unadjusted *p* value of ≤0.05. (**E**) Relative quantification of candidate basal cell-specific miRNAs, miR-184, miR-500, and miR-106b compared to a known basal cell-specific miR-455-3p. Freshly isolated primary human tracheobronchial cells were passaged 3 (P3) and 18 (P18) times in SAGM-EA media (*N* = 3). (**F**) Relative quantification of miRNAs belonging to miR-106b-25 cluster in passage 3 basal cells (*N* = 6). Each dot represents one donor.

To select a miRNA for detargeting experiments, we accessed publicly available data through NCBI Gene Expression Omnibus (GEO) under serial number GSE22145 that compared basal cells vs. columnar cells in nasal airway [14] and found seven miRNAs that were consistently expressed in basal cells but not columnar cells from the nasal epithelia of three donors (Figure 1A). To evaluate the expression of miRNA expression in our cultured human tracheobronchial basal cells expanded in SAGM-EA [9], we used a TaqMan low-density array (TLDA; Applied Biosystems) to quantify relative expression of 377 miRNAs (Supplemental Table S1). Expression of 252 miRNAs was consistently detected in basal cells at passage 3 and at passage 18 (Figure 1B). Of these miRNAs, only nine changed significantly between passage 3 and 18 (FDR test with Q = 5%) and 171 miRNAs did not exceed a ± 2-fold change in expression in the passage (Figure 1C). Using a less stringent test, expression of 15 miRNAs changed significantly between passage 3 and 18 (unadjusted *t*-test *p* ≤ 0.05 and absolute fold change ≥ 2) (Figure 1D).

Comparison of our array data with the nasal miRNA sequencing study demonstrated that miR-106b was one of the few miRNAs that was not expressed in columnar cells. Other miRNAs that were basal cell-specific in the nasal study included miR-184 and miR-500. miR-500 was detected at lower levels than miR-106b in our array study and miR-184 was undetectable. In this regard, miR-106b appeared to be the ideal miRNA to use in basal cell detargeting. We decided that our candidate miRNA should have a higher expression level than that of miR-455-3p, which has been reported to effectively inhibit MUC1 in human epithelial basal cells [15]. To more quantitively evaluate the expression of miR-106 in reference to low (miR-500) and very low (miR-184) basal cell expressing miRNAs, we performed single-plex qPCR for these miRNAs in comparison to that of miR-455-3p (Figure 1E). As expected, the expression levels of miR-184 expression was very low and miR-500a was absent, while miR-106b was more than 11-fold higher than the level of miR-455-3p. Moreover, miR-106b was stable on passage, decreasing by only 30% during the 15 passages. These findings confirmed the validity of the array data and suggested miR-106b was a top candidate for basal cell detargeting.

miR-106b, miR-25 and miR-93 belong to the miR-106b-25 cluster that is located in the 13th intron of mini-chromosome maintenance complex component 7 gene (*MCM7*) [16,17]. We prepared miRNA samples from six donors and analyzed the relative expression of these miRNAs (Figure 1F). The expression levels were fairly consistent between the six random donor samples. Notably, although these miRNAs are in the same cistron, their expression varied over a 10-fold range in airway basal cells (Figure 1F), and the pattern of expression of each of the three miRNAs was also different than that reported for the miR-106b-25 cluster in other tissues [18,19,20,21,22]. Although miR-93 was expressed at ~4.5-fold higher levels than miR-106b, we chose to move forward with miR-106b since miR-93 was observed to be expressed in differentiated human nasal columnar cells [14] (GEO dataset: GSE22145). 

### 3.2. Increasing the Production Yield of a Lentiviral Vector Harboring Bidirectional Expression Cassettes

**Figure 2:**
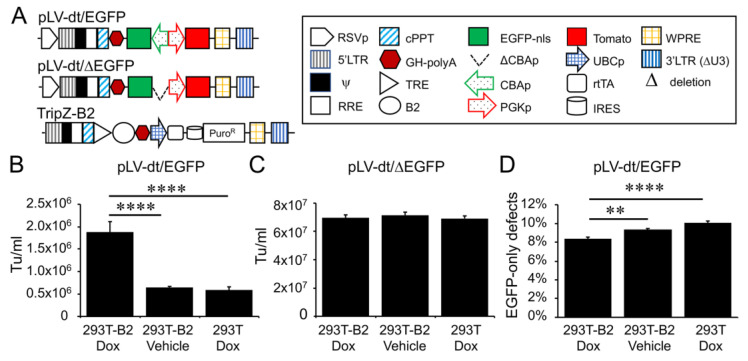
Suppressor of RNAi B2 protein increases titer and viral genome integrity of lentiviral vectors harboring bidirectional gene expression cassettes. (**A**) pLV-dt/ΔEGFP and pLV-dt/EGFP are the proviral vector plasmids used in production of these lentiviral vectors. TripZ-B2 is a lentiviral vector used to make the 293T-B2 cell line that expresses B2 following doxycycline treatment. The box legend to the right highlights the components of these proviral plasmids. Definitions are as follows: 5’LTR, 5’ long terminal repeat; ψ, psi, viral packaging signal sequence; RRE, rev response element, where Rev protein binds; cPPT, central polypurine tract, recognition site for proviral DNA synthesis; STOP, translation stop sequence; EGFP-nls, *EGFP* with nuclear localization signal; ΔCBAp, deletion of chicken beta-actin promoter (CBA); CBAp, CBA promoter in reverse orientation to the viral genomic transcript; PGKp, mouse phosphoglycerate kinase 1 promoter; TRE, tetracycline response element; UBCp, ubiquitin C promoter; rtTA, reverse tetracycline trans-activator; IRES, internal ribosomal entry site; WPRE, Woodchuck hepatitis virus post-transcriptional regulatory element for increasing nuclear export; 3’LTR (ΔU3), 3’ long terminal repeat with deletion in unique 3’ sequence that is necessary for activating viral genome transcription. (**B**) Comparison of LV-dt/EGFP titers produced by 293T-B2 with doxycycline treatment, 293T-B2 with vehicle treatment, and 293T with doxycycline treatment. Data show the mean+/-SEM for *N* = 6 viral preparations. (**C**) Comparison of lentiviral vector LV-dt/ΔEGFP transduction unit per milliliter (Tu/mL) produced by 293T-B2 with doxycycline treatment, 293T-B2 with vehicle (water) treatment, and 293T with doxycycline treatment. Data show the mean+/-SEM for *N* = 6 viral preparations. (**D**) Comparison of the percentage of cells positive for EGFP only among LV-dt/EGFP infected cells produced by 293T-B2 with doxycycline treatment, 293T-B2 with vehicle treatment, and 293T with doxycycline treatment. Data show the mean+/-SEM for *N* = 6 viral preparations. (**B,C**) Statistical comparisons were made by one-way ANOVA, Bonferroni’s multiple comparison test. ****, *p* < 0.0001. **, *p* < 0.01.

In order to evaluate detargeting using a basal cell-specific miR-target (miRT) site, we sought to have two reporter genes (one detargeted and one constitutively expressed) within the lentiviral vector. Since the miRT must reside in the 3′ UTR of the targeted gene cassette, creating this vector required two transgene cassettes (each with unique promoters and 3′ UTRs) oriented in the opposite direction (Figure 2A). We chose a nuclear-targeted EGFP (EGFP-nls) and Tomato as the two transgenes, with the miRT harbored in the 3′ UTR of the EGFP-nls cassette in the reverse orientation. The Tomato transgene in the direct orientation utilized the 3′-LTR polyA site and could not accommodate a miRT without compromising the viral packaging. This vector platform, we call LV-dt/EGFP, was constructed to allow the flexible insertion of any miRT sequence for specific cell type detargeting of transgene expression. 

Initial attempts to generate the LV-dt/EGFP virus gave rise to low titers, despite lacking miRT sequences. We hypothesized complementarity of antisense EGFP mRNA, expressed from the proviral plasmid following transfection, with the full-length sense-strand viral RNA genome might activate RNAi and degrade viral genomes prior to packaging. To approach this problem, we sought to suppress RNAi during packaging with the flock house virus protein B2, which is a known RNAi suppressor [23,24,25]. When a B2-expression plasmid was co-transfected when making LV-dt/EGFP, the resulting virus titer was ~3 times higher than that without B2 (data not shown). We then used a lentivector to stably integrate a *B2* gene expression cassette into 293T cells, however, persistent *B2* expression in 293T cells was toxic. Thus, we generated a 293T cell line that expresses a doxycycline inducible (Tet-on) B2 protein using a TRIPZ vector (Figure 2A). We first tested different concentrations of doxycycline and two time points of doxycycline addition, at the time of proviral vector transfection or at the first media change after transfection. We observed that addition of 500 ng/mL doxycycline at the time of transfection produced highest virus titer while maintaining health of the producer cells. *B2* mRNA induction by doxycycline was verified by qPCR (data not shown). Indeed, the lentiviral vector LV-dt/EGFP titer was significantly increased by doxycycline induced B2 expression during the virus production (Figure 2B). To confirm that the mechanism of reduced titers of LV-dt/EGFP was due to antisense EGFP transcripts, we created a second control vector (LV-dt/ΔEGFP) which lacked the CBA-promoter controlling EGFP expression. Titers of LV-dt/ΔEGFP, which lacked expression of EGFP transcripts with complementary to the viral genome, were not affected by the induction of B2 (Figure 2C).

To calculate viral titers in the above experiments, we used titration transduction assays on 293 cells followed by flow cytometry. We noticed that there were LV-dt/EGFP transduced cells that were positive for only EGFP or Tomato. This suggested that mutations or deletions within the proviral genomes likely occurred prior to packaging. During reverse transcription, a reverse transcriptase may change its templates 8 to 10 times [26] contributing to diversity of the lentivirus in the wild. This is a drawback to lentiviral vectors. We sought to evaluate whether inhibiting RNAi pathways with B2 would improve integrity of the packaged LV-dt/EGFP genomes. To this end, we compared the percentage of LV-dt/EGFP transduced 293T cells that only expressed EGFP (defective particles) from three types of viral preparation conditions: 1) 293T-B2 cells induced with doxycycline, 2) 293T-B2 cell not induced with doxycycline, and 3) 293T cells induced with doxycycline. Results from these flow cytometry comparisons demonstrated that group-1 and group-2 had ~20% and ~10% fewer defective particles than group-3, respectively (Figure 2D). We hypothesize that low level expression of B2 in the uninduced group-2 viral preparations improved integrity of the viral genomes when compared to 293T preparations lacking B2 (group-3).

### 3.3. Detargeting EGFP Expression in Proliferating Basal Cells.

**Figure 3:**
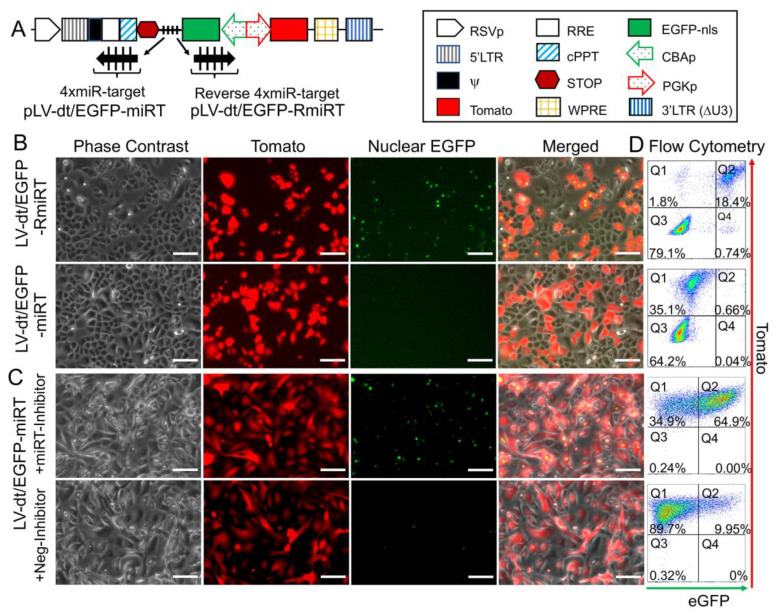
Incorporation of miR-106b target sequence (miRT) into the 3′-UTR of EGFP effectively detargets lentiviral-mediated expression in proliferating basal cells. (**A**) Diagram of the bidirectional promoter proviral lentiviral plasmids (pLV-dt/EGFP-miRT and pLV-dt/EGFP-RmiRT) used to generate lentivirus and test detargeting in basal cells. The box legend to the right highlights the components of these proviral plasmids as described in detail within the Figure 2A legend. LV-dt/EGFP-miRT is the experimental vector harboring a CBA promoter driven nuclear targeted EGFP (EGFP-nls) with miR-106b target sequence (4× miR target or mirT) in the reverse orientation. In the forward direction, the PGK promoter drives expression of the Tomato reporter, which is unaffected by miR-106b. LV-dt/EGFP-RmiRT is a control vector with the miRNA target sequence in the reverse orientation. (**B**) LV-dt/EGFP-miRT and LV-dt/EGFP-RmiRT viruses were used to transduce primary human airway basal cell in SAGM-EA cultures. The Tomato-positive (red) cells indicate the virally transduced cells. EGFP expression is seen in dt/EGFP-RmiRT control transduced cells but not in cells transduced with the detargeted LV-dt/EGFP-miRT vector. Scale bar, 100 µm. (**C**) Basal cells transduced with LV-dt/EGFP-miRT vector, and FACS isolated for Tomato-positive cells, were transfected with miRT-106b inhibitor sequences to block detargeting or mock transfected. Scale bar, 100 µm. (**D**) The cells in (B and C) were analyzed by flow cytometer and are shown in dot plots to the right of the corresponding images for each condition. The percentage of cells are indicated in each quadrant: Q1 (Tomato-positive only cells), Q2 (Tomato and EGFP double-positive cells), Q3 (EGFP-positive only cells), and Q4 (non-fluorescent cells).

To test whether the miR-106b target sequence (miRT) could be used to effectively detarget gene expression in basal cells, we generated a lentivirus vector that contained a nuclear targeted EGFP with the 3′-UTR miR-106b target sequence (pLV-dt/EGFP-miRT) and a control lentivirus vector with the miR-106bT sequence in reverse orientation (pLV-dt/EGFP-RmiRT; Figure 3A). We infected human epithelial basal cells grown in SAGM-EA with LV-dt/EGFP-miRT or LV-dt/EGFP-RmiRT and analyzed EGFP and Tomato expression by flow cytometry and fluorescent imaging. As hypothesized, the nuclei of the LV-dt/EGFP-RmiRT transduced basal cells were EGFP-positive, whereas basal cells transduced with the LV-dt/EGFP-miRT vector were EGFP-negative (Figure 3B). Thus, the miR-106b target sequence in the 3′-UTR appeared to successfully detarget EGFP expression in basal cells. To verify that miR-106b was indeed responsible for *EGFP* knock-down, we transfected FACS isolated Tomato-positive LV-dt/EGFP-miRT transduced cells with a miR-106b inhibitor. As expected, the LV-dt/EGFP-miRT transduced cells transfected with the miR-106b inhibitor recovered nuclear EGFP expression, while the mock transfected negative control cells did not (Figure 3C). The quantification of EGFP-positive only (Q4), Tomato-positive only (Q1), EGFP/Tomato-double-positive (Q2) and double-negative (Q3) cells are shown in the quadrants generated by flow cytometry (Figure 3D).

### 3.4. Basal Cell miRT-106b Detargeting is Partially Maintained in Differentiated ALI Cultures and Organoids

**Figure 4:**
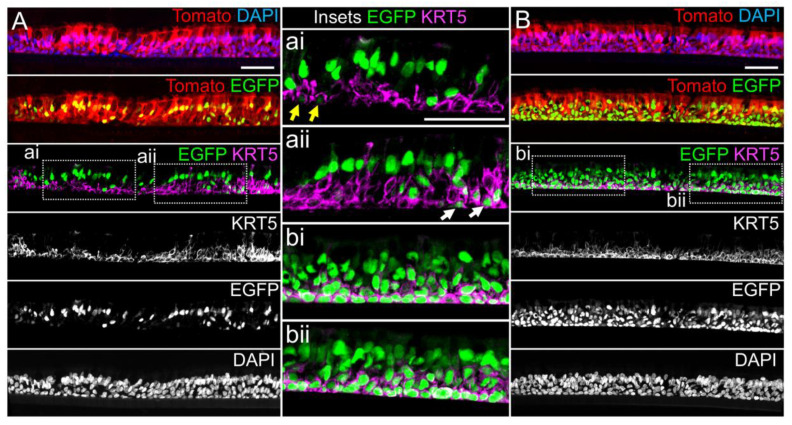
Lentiviral-mediated basal cell detargeting of EGFP following differentiation at an air–liquid interface. Primary human basal cells were transduced with LV-dt/EGFP-miRT or LV-dt/EGFP-RmiRT vectors and expanded for 1–2 days before seeding for differentiation in air–liquid interface (ALI) cultures. (**A,B**) Confocal microscopic images of (**A**) LV-dt/EGFP-miRT and (**B**) LV-dt/EGFP-RmiRT transduced ALI culture using sections immunostained for KRT5 (keratin-5) and imaged for KRT5, EGFP, and Tomato expression with DAPI to mark nuclei. Dual and single channel images are shown. Enlarged boxed regions in (**A**,**B**) are shown in the middle column. Scale bar, 50 μm.

miR-106 is highly expressed in proliferating basal cells grown in SAGM-EA media and basal cell detargeting with miRT-106b is highly effective (Figure 3). To determine if miR-106 expression in basal cells of differentiated cultures was sufficient for detargeting, we studied the EGFP expression profiles of ALI and organoid cultures generated from LV-dt/EGFP-miRT and LV-dt/EGFP-RmiRT transduced basal cells. Approximately 40% of the basal cell population was transduced and the cells were not subjected to FACS prior to making ALI cultures or airway organoids. ALI cultures generated from these two groups were sectioned and evaluated for EGFP, Tomato, and KRT5 (basal cell marker) expression. Both LV-dt/EGFP-miRT and LV-dt/EGFP-RmiRT transduced basal cells formed a pseudostratified epithelium with Tomato expression marking transduced cells and KRT5 marking the basal cell layer (Figure 4). As expected, LV-dt/EGFP-miRT transduced cultures lacked nuclear EGFP expression in the majority of KRT5-positive basal cells, confirming detargeting, but contained EGFP-positive nuclei in differentiated columnar cells (Figure 4A). Conversely, LV-dt/EGFP-RmiRT transduced control cultures contained nuclear EGFP-positive cells throughout the basal layer as well as the columnar cell differentiated layer (Figure 4B). Notably, LV-dt/EGFP-miRT transduced ALI culture lacked EGFP in ~50% of columnar cells, whereas the vast majority of columnar cells expressed EGFP in LV-dt/EGFP-RmiRT cultures. These findings suggest that miR106b may also be expressed or retained in a subset of columnar cells in ALI cultures.

**Figure 5:**
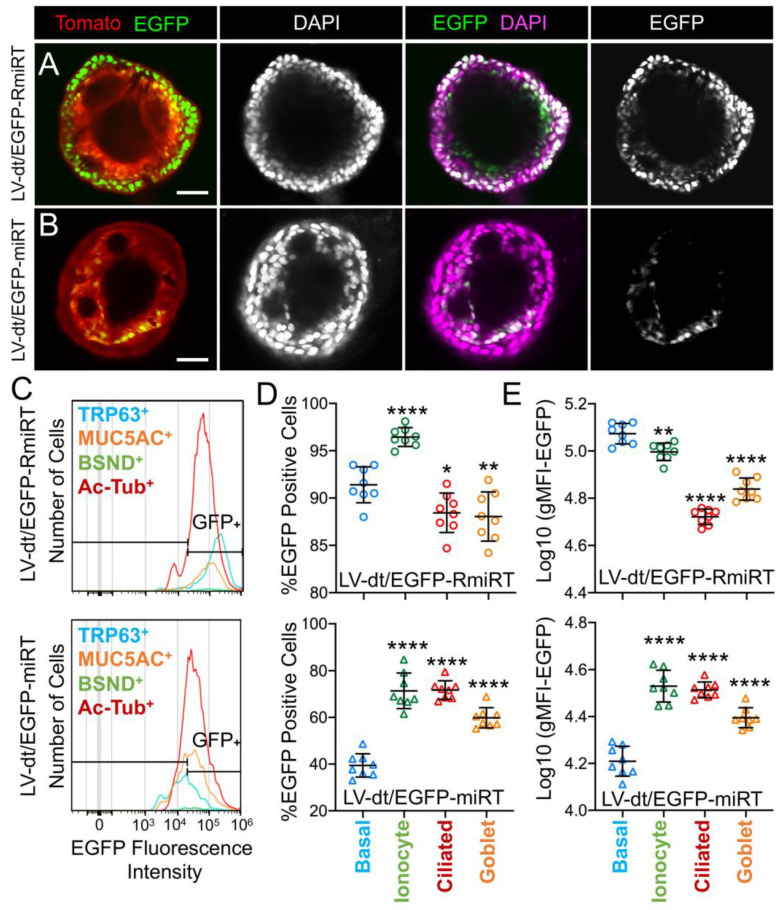
Basal cell detargeting of a reporter transgene in differentiated cell types of ALI cultures. (**A**,**B**) Human basal cells were transduced with (**A**) LV-dt/EGFP-RmiRT or (**B**) LV-dt/EGFP-miRT and expanded for 1–2 days prior to seeding in organoid culture. Confocal microscopic images of live organoids stained with the Hoescht 33342 nuclei marker. Single and dual channel images are pseudocolored to better project nuclear EGFP expression. Scale bar, 50μm. (**C**–**E**) Primary basal cells were transduced with LV-dt/EGFP-RmiRT or LV-dt/EGFP-miRT viruses and then FACS was used to isolated pure Tomato-positive basal cells. These cells were expanded in culture and then seeded into ALI cultures for differentiation and then detached and immunostained for quantification of EGFP expression in various cells types by flow cytometer. (**C**) Epithelial lineages were stained for TRP63/p63 (basal cells; blue), BSND (ionocytes; green), alpha-tubulin (ciliated), and MUC5AC (goblet cells; orange). Representative histogram distributions of lineage-labeled cell populations treated transduced with LV-dt/EGFP-RmiRT (top) or LV-dt/EGFP-miRT (bottom). (**D**) Percentage of EGFP-positive cells for each lineage using the gate shown in (C) which captures 90% of EGFP-positive basal cells in the control LV-dt/EGFP-RmiRT vector group. (**E**) Mean fluorescent intensity (MFI) of lineage-labeled populations. Statistics represent a one-way ANOVA with Dunnett’s multiple comparison test against the basal cell population: * *p* < 0.01, ** *p* < 0.05, **** *p* < 0.0001. Data show the mean +/-SD for *N* = 8 transwells for each condition.

We next performed similar studies in airway organoid cultures generated from LV-dt/EGFP-miRT and LV-dt/EGFP-RmiRT transduced basal cells (Figure 5A). These airway organoids mature with time to form an external basal cell layer and internal luminal cell layer composed of differentiated columnar cells. Similar to ALI cultures, confocal imaging of intact organoids demonstrated that LV-dt/EGFP-RmiRT transduced organoids contained EGFP expressing cells that spanned the outer basal cell layer as well as the differentiated luminal cell layer. By contrast, Tomato-positive LV-dt/EGFP-miRT transduced cells of the organoid lacked EGFP expression in the outer basal cell layer, but EGFP-positive luminal cells were observed. Tomato expression was dimmer than in the outer layer of basal cells in both groups, similar to ALI cultures, suggesting that the PGK promoter may be less active in the basal cells.

To quantify the extent of detargeting in basal cells, we transduced primary human basal cells with LV-dt/EGFP-miRT or LV-dt/EGFP-RmiRT vector systems and FACS isolated Tomato-positive cells for expansion in SAGM-EA prior to seeding into ALI cultures. Well-differentiated ALI cultures were then dissociated, and the single cell suspension of epithelial cells was fixed and stained for markers of basal cells (TRP63), ciliated cells (acetylated tubulin), goblet cells (MUC5AC), and ionocytes (BSND). These populations were then subjected to flow cytometer and the percentages of EGFP-positive cells for each cell phenotype quantified (Figure 5C–E). As expected from confocal imaging of ALI cultures (Figure 4), the fluorescent intensity of all cell types in the LV-dt/EGFP-miRT group was lower than that of LV-dt/EGFP-RmiRT, suggesting that inclusion of the miRT-106b target sequences generally reduces expression of the EGFP transgene. However, quantification of the percentage of EGFP-positive cells demonstrated the largest drop for miRT vs. RmiRT expression in TRP63-positive basal cells (2.3-fold) (Figure 5D). Furthermore, in LV-dt/EGFP-RmiRT transduced cells, the percentage of EGFP-positive basal cells was significantly lower than ciliated and goblet cells, whereas the opposite was observed in LV-dt/EGFP-miRT transduced cells (Figure 5D). Additionally, the mean fluorescent intensity (MFI, calculated as the geometric mean) of EGFP was the highest in basal cells of the LV-dt/EGFP-RmiRT control group, supporting confocal imaging of ALI demonstrating the strongest EGFP expression in KRT5-positive basal cells with this vector (Figure 4B, bi, bii). By contrast, the MFI was the lowest in the LV-dt/EGFP-miRT transduced basal cells as compared to ionocytes, ciliated cells and goblet cells (Figure 5E), similar to those observed histologic studies (Figure 4A, ai, aii). Overall, these findings suggest that the miRT-106b sequences effectively reduce expression of EGFP in basal cells.

An unexpected finding from these cellular phenotyping studies of LV-dt/EGFP-miRT- and LV-dt/EGFP-RmiRT-transduced epithelia was a significant shift in the number of goblet cells and ionocytes (Table 1). The largest shift occurred in the percentage of MUC5AC-positive goblet cells, rising 2-fold (*p* < 0.0001) in LV-dt/EGFP-miRT transduced epithelia as compared to the RmiRT control vector. By contrast, the percentage of ionocytes marginally declined in the LV-dt/EGFP-miRT group (*p* < 0.0388), while the percentage of basal cells and ciliated cells was not significantly different between the two groups. These findings raise the interesting possibility that high-level expression of mRNA containing the miRT sequence could potentially sequester miR-106b and impact processes involved in goblet cell and ionocyte specification.

### 3.5. Basal Cell-Detargeting of CFTR Expression Alters Functional Complementation in CF Airway Epithelia

**Figure 6:**
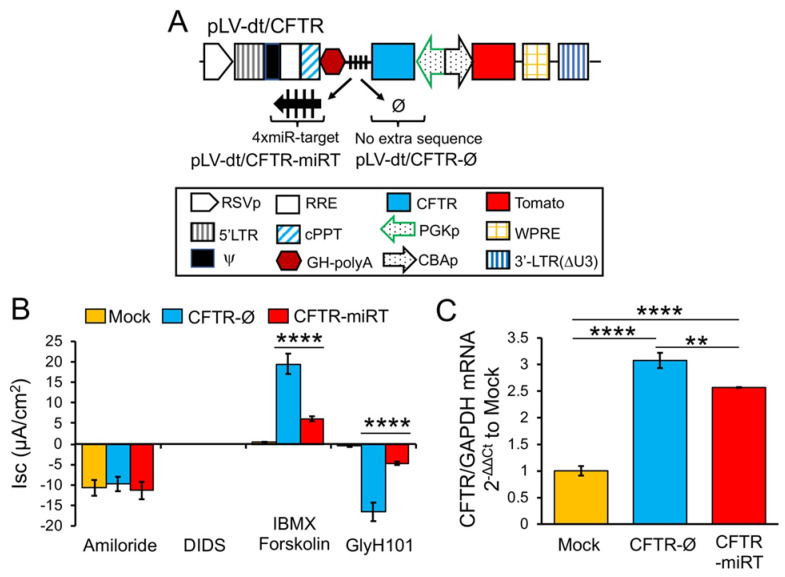
Detargeting CFTR expression in basal cells impacts the level of complementation in CF airway epithelia. (**A**) Diagram of lenti-vector containing CFTR expression cassette in reverse orientation. The PGK promoter (PGKp) drives expression of CFTR with the miR-106b target sequence (4× miR target or mirT) in the 3’UTR. CBA promoter drives expression of Tomato as a reporter gene for viral transduction. pLV-dt/CFTR-Ø is a control vector with no miRT sequence. Box (below) is a legend for each shape in the diagram that highlights the components of these proviral plasmids as described in detail within the Figure 2A legend. (**B**) Short-circuit current (Isc) measurements of differentiated air-liquid interface cultures seeded with transduced at basal cells. Mock, mock-infected cells. PGK-CFTR-Ø, cells transduced by LV-dt/CFTR-Ø; PGK-CFTR-miRT, cells transduced by LV-dt/CFTR-miRT. Amiloride was used to block ENaC-mediated Na^+^ currents. 4,4′-Diisothiocyanatostilbene-2,2′-disulfonic acid (DIDS) was use to inhibit most non-CFTR chloride channel. 3-isobutyl-2-methylxanthine (IBMX) and Forskolin was used for activate CFTR channels. N-(2-naphtalenyl)-(3.5-dibromo-2.4-dihydroxyphenyl)methylene glycine hydrazide (GlyH101) was used to block CFTR. Data show the mean +/-SEM for *N* = 6 transwells for each condition. (**C**) Relative quantification of CFTR mRNA normalized to GAPDH mRNA from each sample used in B. For B and C, the statistics used is one-way ANOVA, Tukey’s multiple comparisons test. ****, *p* < 0.0001. **, *p* = 0.0025. Data show the mean +/-SEM for *N* = 3 independent samples for each condition.

To test our primary hypothesis that detargeting of CFTR in basal cells would improve complementation in CF airway epithelia, we replaced EGFP in LV-dt/EGFP-miRT with CFTR to generate the pLV-dt/CFTR-miRT lentiviral vector. Our control vector (pLV-dt/CFTR-Ø) was identical to pLV-dt/CFTR-miRT but lacked the miR-106b target sequences (Figure 6A). Freshly isolated CF human tracheobronchial basal cells were transduced with each vector and expanded 4 days before seeding into transwells for ALI culture. Contrary to our initial hypothesis, ALI cultures transduced with LV-dt/CFTR-Ø gave rise to ~3.5-fold greater CFTR-mediated CI^–^ currents than that of LV-dt/CFTR-miRT transduced ALI cultures (Figure 6B), even though both cultures expressed similar levels of *CFTR* mRNA, which were 3.2-fold (LV-dt/CFTR-Ø) and 2.7-fold (LV-dt/CFTR-miRT) higher levels than the mock-infected group. Characteristic of CFTR, these currents were induced by cAMP agonists (IBMX/Forskolin) and inhibited by the CFTR channel blocker GlyH101. The slightly lower expression of CFTR mRNA in the LV-dt/CFTR-miRT transduced cultures was expected, consistent with detargeted expression in basal cells. 

## 4. Discussion 

Multipotent basal cells are generally considered the primary stem cell of the large conducting airways [27] and thus are a primary target for stem cell-based genetic therapies for CF. CFTR is expressed in a subpopulation of transitional basal cells (i.e., intermediate basal cells) that are fated to become secretory cells (i.e., goblet cells and club cells) [6,28]. CFTR is also expressed at low levels in a subpopulation of secretory cells and at high levels in pulmonary ionocytes [5,6]. The function of CFTR expression in transitional basal cells remains unclear, but it stands to reason that this expression could be a precursor state to CFTR-expressing daughter cells [3]. The contribution of CFTR expression in secretory cells and ionocytes to the overall level of transepithelial ion transport in airway epithelium is also a source of controversy [3]. Given that CFTR is not expressed in proliferating airway basal cells and the potential that basal cell CFTR expression may impact fate decisions, we reasoned that developing a lentiviral vector that could more closely reproduce endogenous multipotent and transitional basal cell CFTR expression patterns would have utility for CF cell-based therapies. Thus, we sought to develop a lentiviral vector that would repress CFTR expression in proliferating multipotent basal cells and activate CFTR expression in transitional basal cells as they commit to differentiate. 

Gene replacement cell-based therapies for CF will require the expansion of basal cells in conditionally reprogrammed culture and the introduction of a corrected *CFTR* gene using an integrating approach (e.g., lentivirus) [29,30]. It was on this rationale that we designed a lentiviral vector with bi-directional promoters capable of carrying miRT sequences within a heterologous 3’-UTR of the reverse oriented target gene. Through bioinformatics and experimentation, we identified miR-106b as being highly expressed in both proliferating human tracheobronchial and nasal basal cells (Figure 1), but absent in differentiated columnar cells. Thus, the miRT for miR-106b appeared to be suitable for approaching our studies. Notably, the regulated miRT gene must be placed in the reverse orientation since a heterologous UTR in the direct orientation would prematurely terminate the viral genomic RNA during virus production [31]. Lentiviruses with reverse-oriented expression cassettes produce low viral titers due to a double-stranded RNA response and cleavage of the vector RNA genome by cellular Dicer [31,32]. However, previous attempts have successfully produced high titer virus when an inducible promoter is used to drive the reverse-oriented gene of interest [31]. 

Our studies required the use of a strong promoter for both expression cassettes and, like others, we found titers to be low within our bidirectional pLV-dt/EGFP vector. We improved the inherently low titer using a suppressor of RNA silencing (SRS), B2 protein from flock house virus. Although most viral infections activate RNAi responses in the cell against the virus [33,34], only a few have looked into using RNAi suppressors in animal viral vector production [35,36]. In lentiviruses, potential SRSs include Nef and Tat [37,38]; Tat is included with psPAX2 packaging plasmid and may help protect the lentiviral genome during virus production. By utilizing the B2 protein, pLV-dt/EGFP vector production was further increased 3-fold (Figure 2). In this study, we only used B2, but other SRSs of some well-known plant and animal virus SRSs [33,39,40] are worth investigating and may further improve virus production with a bi-directional vector. One downside of using the 293T-B2 cell line is that the cells seem to be even less tightly attached to the surface of the culture dish than the wild-type 293T, so when adding transfection reagents or media it should be carried out very carefully. To mitigate this problem, poly-lysine coating the culture dish may help the cells to attach more tightly and help improve the virus titer. 

Our studies evaluating miR-106b-mediating detargeting using the pLV-dt/EGFP-miRT vector system demonstrated robust shut-off of the EGFP-miRT reporter in proliferating basal cells (Figure 3). Adjusting for differences in functional titer, the miRT-106b reduced EGFP expression 54-fold in proliferating human basal cells as compared to the RmiRT-106b control vector. However, when the pLV-dt/EGFP-miRT basal cells were differentiated at an ALI, there was a subset of basal cells that were not detargeted and a subset of columnar cells that were detargeted (Figure 4). We do not know whether quiescent G0 and intermediate basal cells express miR-106b and this could impact detargeting of pLV-dt/EGFP-miRT in KRT5-positive basal cells. The finding of miRT-106b detargeted columnar cells also suggests that at least in differentiated ALI culture systems, miR-106b expression is expressed or retained in a larger subset of columnar cells than previously observed in human nasal epithelia [14]. Culture conditions likely impacted miR-106b expression and the level of detargeting since organoid cultures demonstrated robust basal cell detargeting with the pLV-dt/EGFP-miRT as compared to the RmiRT-106b control vector. It is also worth noting that in ALI cultures the CBA promoter used to drive EGFP expression in the pLV-dt/EGFP is robustly expressed in basal cells, while the PGK promoter used to drive Tomato expression is more active in columnar cells and less active in basal cells (Figure 4). These differences were even more greatly accentuated in organoid cultures (Figure 5A,B) where very weak EGFP expression was observed in the luminal cell layer for both vector systems.

Due to the relative activity of the CBA and PGK promoters in basal vs. columnar cells of ALI cultures and organoids, we altered the sequence of the promoters in our pLV-dt/CFTR-miRT and pLV-dt/CFTR-Ø vector systems. Since the PGK promoter was weaker in basal cells and stronger in columnar cells, it was used to drive CFTR expression, thus accentuating the detargeting by miRT-106b in basal cells and facilitating CFTR expression in differentiated cells that participate in ion transport (Figure 6A). Similarly, the CBA promoter had greater expression in basal cells and thus was used to drive Tomato expression. Contrary to our original hypothesis, CF ALI cultures transduced with LV-dt/CFTR-miRT had significantly less functional correction of CFTR currents as compared to LV-dt/CFTR-Ø (Figure 6B). 

While the reason for the observed difference in CFTR complementation is currently unknown, there are three potential explanations that warrant further investigation. First, LV-dt/EGFP-miRT transduced ALI cultures had a subpopulation of columnar cells that were Tomato-positive and EGFP-negative. This phenotype was rarely observed in control ALI transduced with the pLV-dt/EGFP-RmiRT vector. Thus, these results would be consistent with expression of miR-106b in a subset of columnar cells that contribute to CFTR-mediated current.

Second, expression of the EGFP-miRT-106b transcript in basal cells led to significant changes in two cell populations when differentiated at ALI (i.e., ionocytes and goblet cells) (Table 1). The decrease in the percentage in ionocytes was relatively small (12%), while the increase in goblet cells was large (200%). Both of these cellular compartments express CFTR in a subpopulation of each cell type. One possibility for this vector-related shift in differentiated cell types is that high-level expression of EGFP-miRT-106b transcripts may sequester miR-106b and act like a miR-inhibitor. Thus, it is possible that inhibiting miR-106b activates differentiation toward columnar cells that cannot participate in CFTR-mediated ion transport. This possibility can be supported by two interpretations of CFTR mRNA levels shown in Figure 6C, which are not mutually exclusive. The mild reduction in total CFTR mRNA levels in LV-dt/CFTR-miRT transduced epithelia is consistent with successful CFTR detargeting in basal cells, where the decrease may represent expression of CFTR in basal cells. However, we cannot rule out that inhibiting miR-106b may have led to an expansion of cell types that cannot facilitate CFTR-mediated anion transport, and that we cannot currently account for with the flow cytometry panel described. Future studies using single-cell RNAseq could help to understand the shift in cellular compartments and their *CFTR* expression patterns. 

The last formal possibility for explaining these results, however unlikely, is the contribution of basal cell CFTR expression to transepithelial anion transport. Current wisdom suggests that only channels that reside in the apical and basolateral membranes of polarized epithelia contribute to transepithelial ion movement. However, very little is known about why CFTR is expressed in basal cells, so we cannot rule this out as a formal possibility.

miR-106b is one of the three miRNAs in the polycistronic miR-106b ~25 cluster within an intron of the *MCM7* gene. MCM7 is part of the DNA replication initiation complex, but its expression is not necessarily coupled to that of miR-106b [19,20]. miR-106b can also play roles in cell-cycle regulation of both stem cells and cancer cells [16,41]. For example, expression of this miRNA enhances cell growth [42], promotes migration of certain cancer cells [43], and promotes cell cycle progression [44]. In SAGM-EA conditionally reprogramming media, basal cells are locked into a self-renewing state. However, it remains unclear if miR-106b plays a role in cell cycle progression of basal cells cultured under these conditions. We did not observe a major difference in morphology or growth of cells expressing the miRT-106b target in either EGFP or CFTR transcripts, and this might suggest that sequestration of miR-106b from its native targets does not occur if these biologic functions of miR-106b are relevant to airway basal cells.

## 5. Conclusions

This study has strengths and limitations. One limitation includes a clearer understanding of cellular expression patterns of miR-106b in ALI cultures. While we had initial chosen miR-106b as a candidate based on its lack of expression in nasal columnar cells, our reporter gene expression studies suggest that it may be expressed in a subset of columnar cells. Future studies evaluating miR-106b expression in FACS-isolated cell types would allow for a clearer interpretation of how the miRT-106b sequence alters CFTR complementation. A second limitation is the fact the promoters used in the bicistronic vectors studied have slightly different activities in basal vs. columnar cells. The use of a bidirectional promoter might be a better approach for future studies, however, the most commonly used major immediate-early cytomegaloviruses enhancer/promoter is typically inactivated in lentiviral vectors by methylation. A strength of these studies includes the development of miRT-106b vectors that can clearly detarget expression in proliferating basal cells. Such a vector system can be used to study basal cell differentiation through the regulated expression of transcription factors that would otherwise terminally differentiate proliferating basal cells. A second strength includes the novel findings that miR-106b appears to be expressed in specific populations of cells that contribute to CFTR-mediated transepithelial ion transport and/or that certain cell types can express CFTR mRNA but not participate in CFTR-dependent transepithelial anion transport. Although more research is needed to understand the mechanism, the finding itself has implications for CF gene therapy as it implies unique cellular targets for CFTR complementation. 

## Figures and Tables

**Table 1:** Distribution of cell types in differentiated ALI cultures.

Vector	% Basal Cells (TRP63+)	% Ionocytes (BSND+)	% Ciliated Cells (Ac-Tubulin+)	% Goblet Cells (MUC5AC+)
LV-dt/EGFP-RmiRT	21.8+/−3.8 *	0.82+/−0.02	44.3+/−1.6	11.2+/−0.8
LV-dt/EGFP-miRT	22.4+/−1.5	0.72+/−0.04	41.5+/−0.9	21.9+/−1.2
*p*-value **	0.7768	0.0388	0.1567	<0.0001

The percentage of viable cells positive for each of the phenotypic cellular markers is shown (TRP63-basal cells; BSND-ionocytes; acetylated tubulin-ciliated cells; MUC5AC-goblet cells). Cells not positive for any of the four antibodies were 21.9% and 12.5% for LV-dt/EGFP-RmiRT- and LV-dt/EGFP-miRT-transduced epithelia, respectively. * Mean +/-SEM. ** Statistical comparisons by Welch’s *t* test.

## Supplementary Materials

The following are available online at https://www.mdpi.com/2073-4425/11/10/1169/s1, Table S1: PCR array table.

## Abbreviations

CF	cystic fibrosis
CFTR	cystic fibrosis transmembrane conductance regulator
miR	microRNA
3’-UTR	3’-untranslated region
PGK	mouse phosphoglycerate kinase 1 promoter
CBA	CMV early enhancer-chicken beta-actin promoter
Tomato	dTomato, dt
SAGM-EA	Small Airway Epithelial Growth Medium with extra additives
